# Lymphovascular invasion is a high risk factor for stage I/II colorectal cancer: a systematic review and meta-analysis

**DOI:** 10.18632/oncotarget.15425

**Published:** 2017-02-16

**Authors:** Hang Yuan, Quanjin Dong, Bo'an Zheng, Xinye Hu, Jian-Bo Xu, Shiliang Tu

**Affiliations:** ^1^ The Surgical Department of Coloproctology, Zhejiang Provincial People's Hospital, Hangzhou, China; ^2^ Nanjing Medical University, Nanjing, China; ^3^ Department of Hepatobiliary Surgery, Huai'an First People's Hospital, Nanjing Medical University, Huai'an City, China

**Keywords:** lymphovascular invasion, survival, stage I/II, colorectal cancer, meta-analysis

## Abstract

The prognostic value of lymphovascular invasion (LVI) in stage I/II colorectal cancer (CRC) does not reach a consensus. To systematically assess prognostic significance of LVI, databases of PubMed, Web of Science, and Embase were searched from inception up to 10 Dec 2016. The pooled hazard ratio (HR) and 95% confidence intervals (CI) were used to determine the prognostic effects. Nineteen relevant studies including 9881 total patients were enrolled. Our results showed that LVI is significantly associated with poor prognosis in overall survival (OS) (HR=2.15, 95 % CI=1.72–2.68, *P* < 0.01) and disease-free survival (DFS) (HR=1.73, 95% CI=1.50–1.99, *P* < 0.01), which is similar in stage II patients. Further subgroup analysis revealed that the significance of the association between LVI and worse prognosis in CRC patients is not affected by below factors, including geographic setting, LVI positive rate, treatment, tumor site, and quality of the study. The current meta-analysis suggests that LVI may be a poor prognostic factor for stage I/II CRC patients.

## INTRODUCTION

Colorectal cancer (CRC) is one of the leading malignant diseases worldwide. As in other types of cancer, the lymphatic system is the primary pathway of metastasis for CRC. Lymph node status is commonly used to identify a patient's prognosis, tumor stage, and treatment modality [1]. Patients without lymph node metastasis are classified as UICC stage I or II, depending on the infiltration depth. These patients have a favorable prognosis, and adjuvant chemotherapy is restricted to particular risk situations [2]. Nevertheless, approximately 10% to 20% of colon cancer cases show an adverse clinical course. To date, there is no generally accepted diagnostic tool available that could predict which of those cases are vulnerable to developing progressive disease.

Lymphovascular invasion (LVI) is thought to be involved the progress of lymphatic metastasis. The National Comprehensive Cancer Network (NCCN) Guidelines defined several additional factors including LVI status to identify patients at an increased risk for progressive disease in stage II colorectal cancers [2]. However, it remains unclear whether lymphovascular invasion marks a poor prognosis for patients with CRC. Although some researchers have found that patients with LVI positive tumors have a worse prognosis than those with LVI negative tumors [3–5], other investigators have reported that LVI is of no prognostic significance [6, 7]. It would be of great value to detect whether LVI is associated with a worse prognosis, to be a supplement to existing staging systems to determine whether a patient is suitable for adjuvant treatment [8]. The present study was designed to systematically assess the association between LVI and the prognosis of early stage (stage I/II) CRC patients.

## RESULTS

### Search results

**Figure 1:**
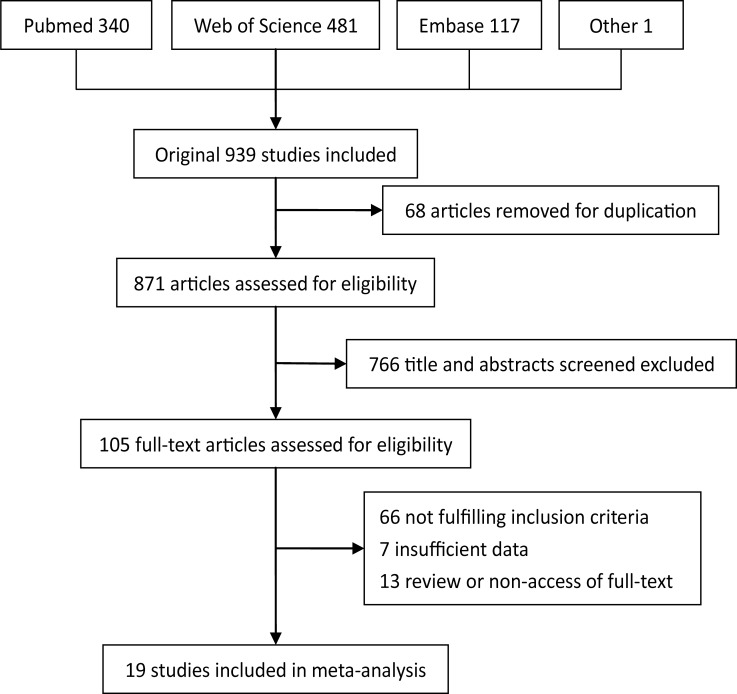
Flow diagram of literature search and study selection

A total of 939 studies were retrieved from the database search, of which 513 studies were excluded as duplicates, 557 as inappropriate publication types, insufficient data or unrelated to stage I/II CRC. 79 full-text publications were left over to assess the eligibility. One study failed to get full-text was included due to a great number of sample size and sufficient data calculated from abstract [9]. Eventually, 19 articles met the inclusion criteria and were included in the analysis [7, 9–26]. Figure 1 demonstrates the detailed process of articles identification and selection.

### Baseline characteristics of included studies

Overall, 9881 stage I/II CRC patients were included. A summary of the characteristics of all included studies were exhibited in Table 1. Among the included 19 articles with sample sizes ranging from 82 to 2649 patients (median = 341), 9 studies (4068 patients, 41.17 %) were based on Asian populations; the remaining 10 studies (5813 patients, 58.83 %) were performed in non-Asian populations. The incidence of lymphovascular invasion ranges from 5.2% to 30.3%, and it was significantly higher in non-Asian region than in Asian region (*P* < 0.05). Nine studies included colon cancer [13–16], 3 included rectal cancer and 7 included total colorectal cancer (without distinguishing colon and rectal cancer) [13–16]. The study quality scores, evaluated by the NOS, ranged from 5 to 8 (with a mean of 6.3), except one study that could not be analyzed because of only access to abstract [9]. Nine studies reported OS and 13 reported DFS as the prognostic indicator.

**Table 1:** demonstrates the detailed process of articles identification and selection

Author	Published year	Region	Number (male/female)	Age mean±SD/ median (range)	Follow up(m) mean±SD/ median (range)	Tumor site	Number LVI+ (%)	nCRT	pCRT	TNM stage	Outcome	SQ^a^
Ghosh	2016	Australia	690(393/297)	NR	53.5(34-65)	C	209(30.3)	NR	NR	II	DFS	5
Nikberg	2016	Sweden	2649(NA)	NA	NA	R	387(14.6)	P	P	II	DFS	NA
Zhang	2016	China	333(188/145)	63(17-86)	52.23±29.7	C	40(12.0)	N	P	II	OS	8
Peng	2014	Australia	458(252/206)	73(23-97)	62.4(1.3-126)	C	115(25.1)	N	P	II	OS	8
Patel	2014	US	175(95/80)	65(24-89)	720	R	24(13.7)	N	N	I	OS	6
Du	2014	China	145(84/61)	69(21-82)	68.5(6-120)	C	10(6.9)	N	N	I/II	DFS	8
Lin	2014	Taiwan	962(612/350)	71.8(24-107)	60.2(4-106)	CR	50(5.2)	N	N	II	DFS	7
Artac	2014	Turkey	554(332/222)	62(26-88)	NR	C	107(19.3)	N	P	II	DFS	7
Venook	2013	US	690(360/330)	NR	NR	C	78(11.3)	NR	P	II	DFS	5
Betge	2012	Austria	120(61/59)	71.2(33.4-85.2)	83(1-180)	CR	26(21.7)	N	NR	II	OS/DFS	7
Barresi	2012	Italy	82(45/37)	70(48-89)	NR	CR	23(28.0)	N	N	I	OS	5
Choi	2010	Hong Kong	664(385/279)	70(27-96)	44(12-104)	CR	88(13.3)	NR	P	II	DFS	7
Lim	2010	Korea	903(NR)	NR	87.5(3-120)	CR	95(10.5)	N	P	II	OS/DFS	6
Huh	2010	Korea	341(209/132)	63.1(22-85)	57.6(0.4-106.2)	CR	44(12.9)	N	P	II	DFS	6
Lin CC	2009	Taiwan	375(274/101)	68.3±12.1	48.5(0.7-96.6)	C	22(5.9)	NR	P	II	DFS	7
Earle	2009	US	258(139/119)	NR	NR	C	63(24.4)	N	P	II	OS	6
Lee	2006	Korea	121(89/32)	57.7(28-80)	NR	CR	25(12.9)	NR	P	I/II	DFS	6
Law	2005	Hong Kong	224(141/83)	69(27-89)	NR	R	29(12.9)	NR	NR	II	OS/DFS	6
Lennon	2003	US	137(79/39)	70(36-90)	72(36-108)	C	34(24.8)	NR	NR	II	OS	4

AJCC American Joint Committee on Cancer, R rectum, C colon, CR colorectum, DFS disease-free survival, N none of patients accept the therapy, nCRT neoadjuvant chemoradiotherapy, NR not reported, NA not access, OS overall survival, P part of patients accept the therapy, pCRT postoperative chemoradiotherapy, LVI+ the colorectal cancer patient with lymphovascular invasion positive, SD standard deviation, SQ score of study quality,

^a^ Study quality was judged based on the Newcastle-Ottawa Scale

### Data analysis

#### LVI and OS in CRC

**Figure 2:**
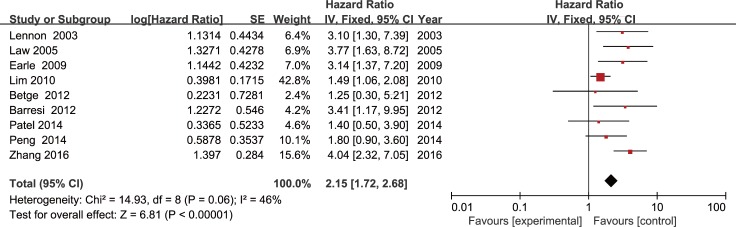
Forest plot of the hazard ratio for the association of lymphovascular invasion with overall survival in colorectal cancer patients

**Figure 3:**
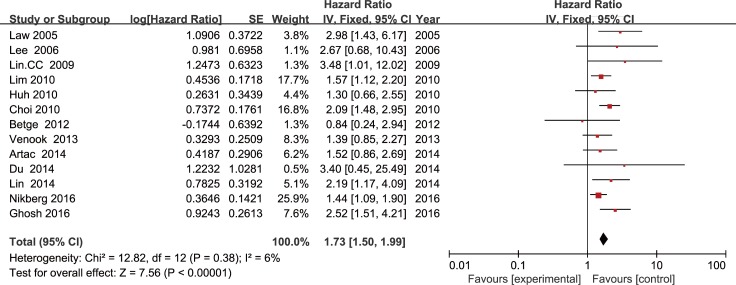
Forest plot of the hazard ratio for the association of lymphovascular invasion with disease free survival in colorectal cancer patients

A meta-analysis of 9 studies on OS demonstrated that LVI positive is associated with poor prognosis in CRC patients with stage I/II (HR = 2.15, 95 % CI = 1.72-2.68, *P* < 0.01; Figure 2) using a fixed-effect model for no significant heterogeneity observed (*I*2 = 46 %, *P* = 0.06). DFS data were calculated from 13 studies by the fixed-effect model. Pooled analysis showed a significant association between LVI and DFS (HR = 1.73, 95% CI = 1.50-1.99, *P* < 0.01; Figure 3) with no significant heterogeneity observed (*I*2 = 6%, *P* = 0.38).

To distinguish between stage I and II, subgroup analysis by stage of TNM was conducted. In stage I group, only OS data were available. The result showed LVI positive was not associated with poor OS using a random-effect model (HR = 2.16, 95 % CI: 0.90-5.16, *P* = 0.08). However, LVI predicted poor OS and DFS in stage II group using a random-effect model (OS: HR = 2.45, 95 % CI: 1.65-3.64, *P* < 0.01; DFS: HR = 1.71, 95 % CI: 1.48-1.98, *P* < 0.01).

Furthermore we performed other subgroup analysis by district (Asian *vs*. non-Asian patients), LVI positive rate (≤15 *vs*. > 15 %), sample sizes (≤190 *vs*. > 200), tumor site (colon *vs*. rectum *vs*. colorectum) and study quality (≤6 *vs*. > 6), neither of which alter the prognostic role of LVI in OS/DFS. (Table 2)

**Table 2:** Results of overall and subgroup analyses for effects of LVI on overall and disease-free survival in colorectal cancer

Categories	*N*	Patients	Pooled HR(95 % CI)	*P* value	Heterogeneity		Model used
					*I*^2^	P*	
Overall survival (OS)	9	2690	2.39(1.70-3.36)	<0.01	46	0.06	FEM
Subgroup 1: Asian	3	1460	2.70(1.27-5.73)	0.01	82	<0.01	REM
Non-Asian	6	1230	2.27(1.56-3.30)	<0.01	0	0.61	REM
Subgroup 2: LVI positive rate >15 %	5	1055	2.45(1.64-3.65)	<0.01	0	0.63	REM
LVI positive rate ≤15 %	4	1635	2.38(1.28-4.43)	<0.01	74	<0.01	REM
Subgroup 3: sample size>200	4	1952	2.33(1.37-3.96)	<0.01	71	0.02	REM
Sample size≤200	5	738	2.63(1.68-4.10)	<0.01	0	0.47	REM
Subgroup 4: colon cancer	4	1186	2.99(2.08-4.31)	<0.01	6	0.36	REM
Rectal cancer	2	399	2.41(0.91-6.32)	0.08	53	0.14	REM
Colorectal cancer	3	1105	1.64(1.10-2.43)	0.02	10	0.33	REM
Subgroup 5:study quality score>6	3	911	2.43(1.22-4.84)	0.01	55	0.11	REM
Study quality score≤6	6	1779	2.32(1.54-3.50)	<0.01	42	0.12	REM
Disease-free survival (DFS)	10	8438	1.73(1.50-1.99)	<0.01	6	0.38	FEM
Subgroup 1: Asian	8	3735	1.92(1.57-2.34)	<0.01	0	0.56	FEM
Non-Asian	5	4703	1.55(1.27-1.90)	<0.01	17	0.30	FEM
Subgroup 2: LVI positive rate >15 %	4	1485	1.91(1.34-2.72)	<0.01	17	0.30	FEM
LVI positive rate ≤15 %	9	6953	1.69(1.45-1.98)	<0.01	9	0.36	FEM
Subgroup 3: sample size>200	10	8052	1.73(1.50-2.00)	<0.01	16	0.29	FEM
Sample size≤200	3	386	1.66(0.72-3.83)	0.24	4	0.35	FEM
Subgroup 4: colon cancer	5	2454	1.86(1.35-2.55)	<0.01	11	0.34	REM
Rectal cancer	2	2873	1.91(0.95-3.82)	0.07	70	0.07	REM
Colorectal cancer	6	3111	1.64(1.39-1.94)	<0.01	0	0.51	REM
Subgroup 5:study quality score>6	5	2266	2.09(1.57-2.77)	<0.01	0	0.57	FEM
Study quality score≤6	7	3523	1.72(1.41-2.11)	<0.01	8	0.37	FEM

FEM fixed-effect model, REM random-effect model, HR hazard ratio, N number of studies, pCRT postoperative chemoradiotherapy, P* *P* value of Q test for heterogeneity test, 95%CI 95%confidence interval, LVI lymphovascular invasion

### Evaluation of heterogeneity

**Figure 4:**
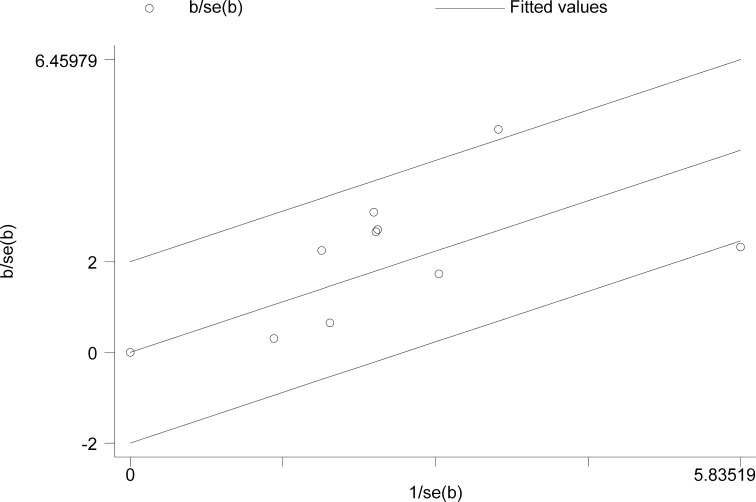
Galbraith plot analysis in overall survival

Because of a relative higher *I*2 value for heterogeneity found in OS among the included studies (*I*2 = 46%, *p* < 0.01), the Galbraith plot test was performed to detect the potential source of heterogeneity. The result demonstrated that there was no specific study could be the major source of heterogeneity (Figure 4).

### Cumulative meta-analysis

**Figure 5:**
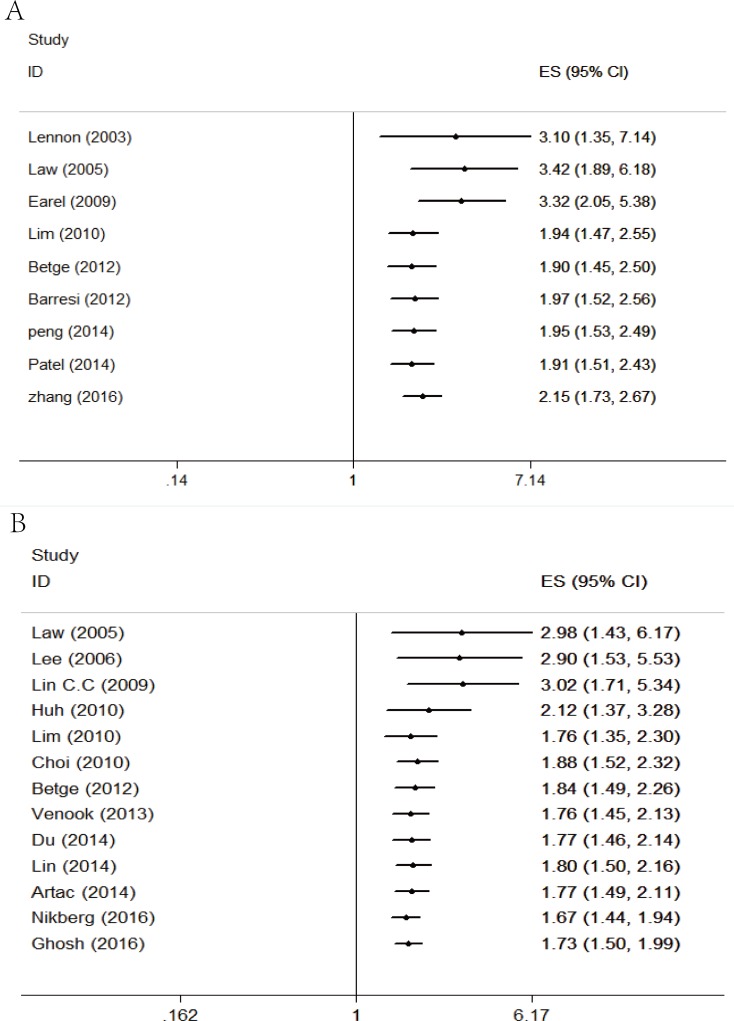
Forest plot of cumulative meta-analysis of the association of lymphovascular invasion with overall survival (**A**) and disease free survival (**B**) in colorectal cancer patients.

Cumulative meta-analysis was performed by ordering the included studies based on publication year. The results of cumulative meta-analysis indicated the correlation between LVI and prognosis of colorectal cancer (OS and DFS) in chronologic order (Figure 5). The 95% CIs have become narrower with increased sample sizes, indicating that the accuracy of the estimates was increasing by the continuous inclusion of studies.

### Sensitivity analysis and publication bias

**Figure 6:**
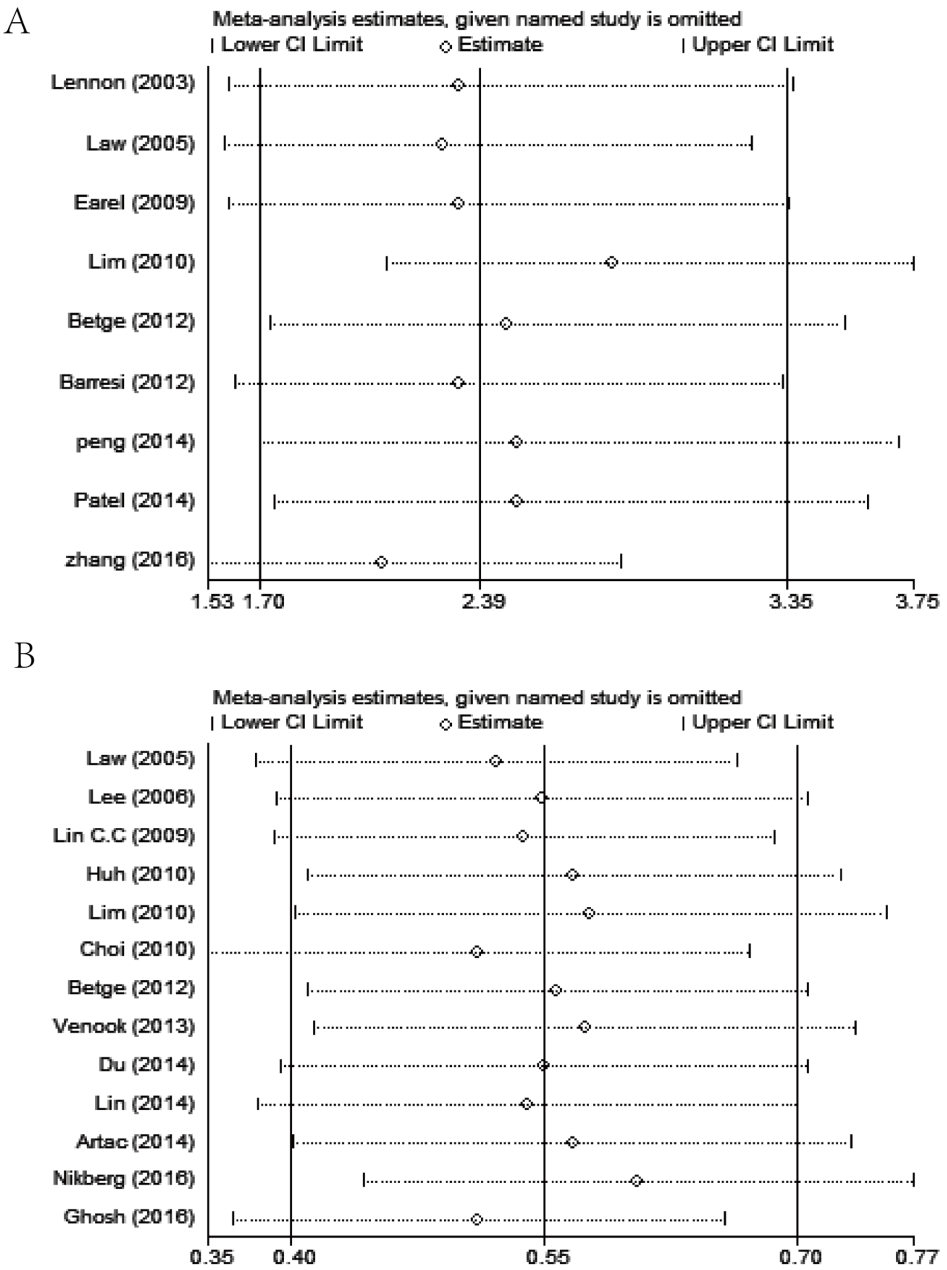
The results of sensitivity analysis of overall survival (**A**) and disease free survival (**B**) showing the effect of each study on the overall estimate by sequentially excluding one study in one turn.

Sensitivity analysis was carried out on OS and DFS to assess the stability of the results by sequentially excluding each study in one turn. In present analysis, no study could possibly affect the pooled risk estimate (Figure 6).

**Figure 7:**
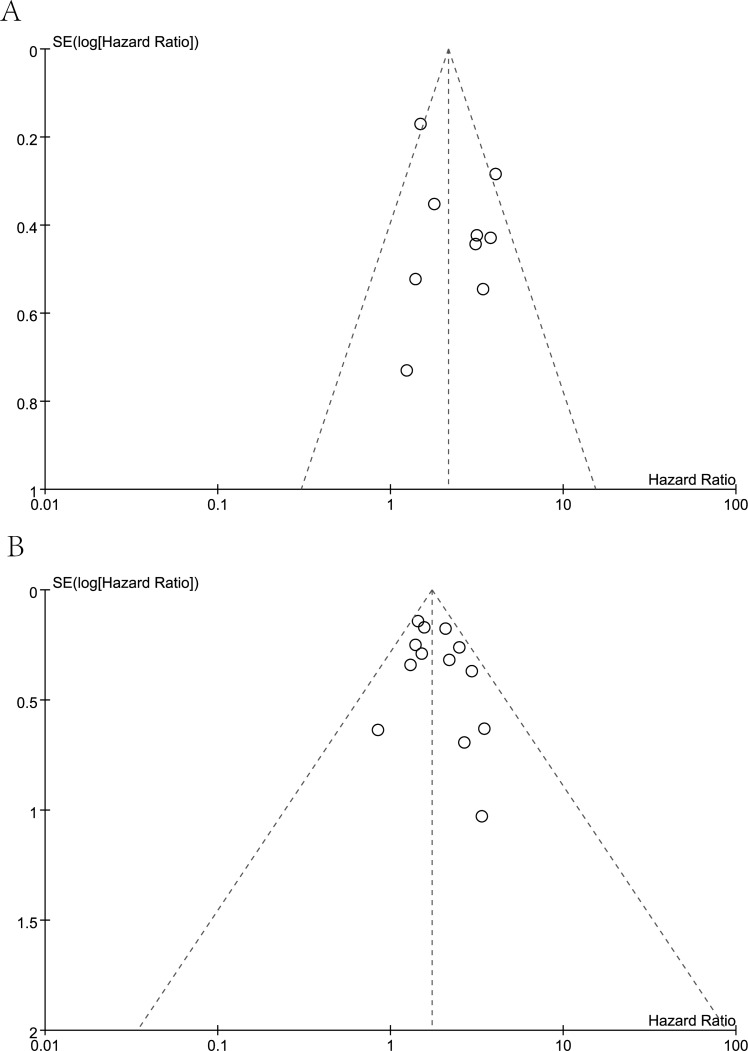
Funnel plot analysis **A**. Funnel plot analysis of 9 studies on overall survival. **B**. Funnel plot analysis of 13 studies on disease free survival.

**Figure 8:**
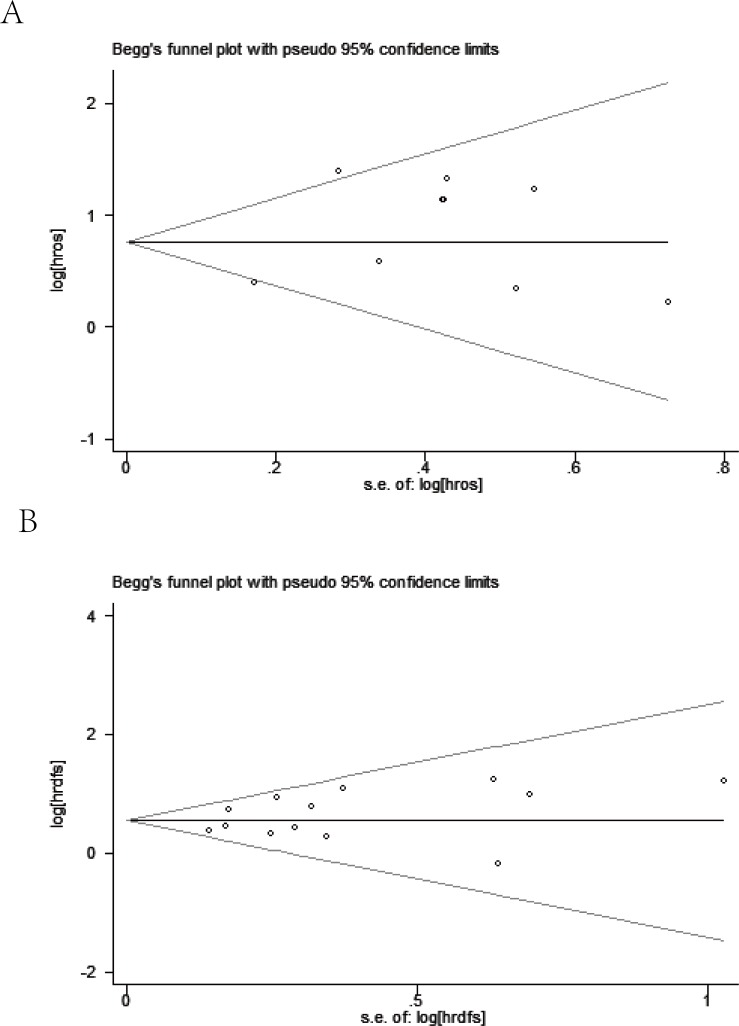
Begg's funnel plot on overall survival (A) and disease free survival (B)

**Figure 9:**
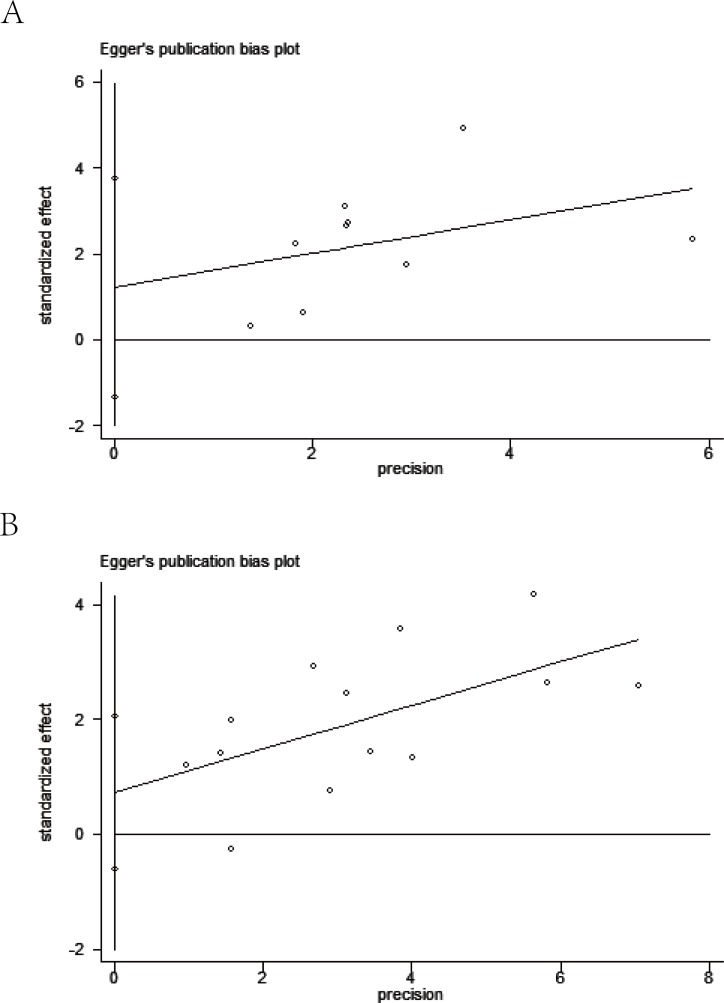
Egger's publication bias plot on overall survival (A) and disease free survival (B)

**Figure 10:**
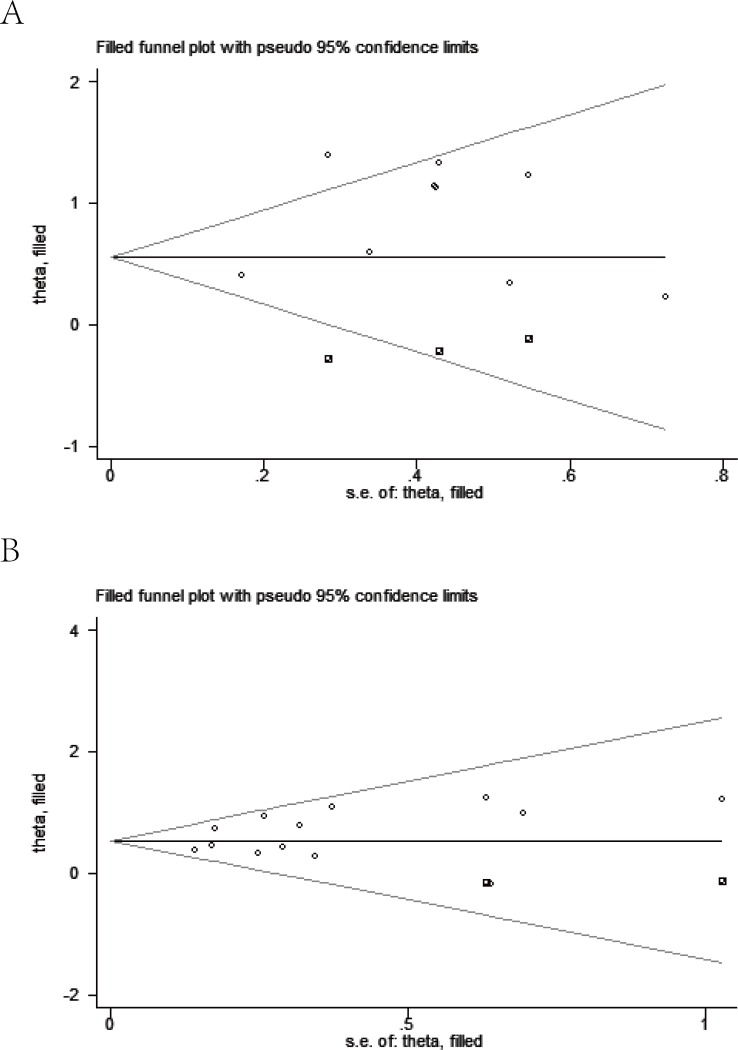
Trim-and-fill funnel plot on overall survival (A) and disease free survival (B)

Meanwhile, visual assessment of funnel plots (Figure 7), as well as Begg's (Figure 8) and Egger's test (Figure 9) on OS and DFS were performed to assess the publication bias of the included researches. No significant asymmetrical distributions were observed in both groups. No evidence of publication bias was detected by Begg's and Egger's test (OS: *P* = 0.754, *P* = 0.291; DFS: *P* = 0.583, *P* = 0.254 respectively). Three possible missing studies in OS group and two in DFS group were identified by the trim-and-fill method using fixed-effect model (Figure 10). These missing studies would not change the trend of the results, so our results were reliable. (OS: HR = 1.742, 95% CI: 1.434-2.115; DFS: HR = 1.705, 95% CI: 1.482-1.961).

## DISCUSSION

Adjuvant chemotherapy remains controversial in early stage (stage I/II) colorectal cancer, especially who would benefit from adjuvant chemotherapy [27, 28]. Uncertainty on the benefit of adjuvant chemotherapy in patients with early stage disease is owing to the fact that which patients at high risk of recurrence are unknown [29]. Hence identification of high-risk stage I/II colorectal cancer patients becomes a clinical concern.

A massive of clinicopathologic features have been associated with a high risk of recurrence and death: tumor stage T4, small number of lymph nodes retrieved, poor differentiation, bowel obstruction, extensive intratumoral necrosis, emergency setting, positive LVI or perineural invasion, and elevated preoperative CEA [30–33]. According to the NCCN guidelines, high risk features for rectal cancer include positive margins, lymphovascular invasion, poorly differentiated tumors, or sm3 invasion. Moreover, poorly differentiated histology, lymphovascular invasion, bowel obstruction, < 12 lymph nodes examined, perineural invasion, localized perforation, or close, indeterminate, or positive margins are considered as high risks for colon cancer(see at www.NCCN.org). From the list above, LVI tends to be an important prognosis predictor after resection of early stage CRC. However, there are no data in high-risk stage II patients that correlate risk features and selection of chemotherapy. The benefit of adjuvant chemotherapy does not improve survival by more than 5% [2, 34]. There is no consensus on the necessity for adjuvant chemotherapy in patients with high-risk stage II cancer [22, 35, 36], whereas most clinicians in China tend to use it for them. In early stage CRC, conflicting study results and insufficient high level evidence regarding the association between LVI and survival data make it necessary to perform a quantitative meta-analysis.

To the best of our knowledge, the present meta-analysis is the first study to provide a system review and meta-analysis on prognostic significance of LVI in early stage (stage I/II) CRC. The included 19 studies and 9881 participants significantly enhanced the statistical power and provided more reliable results. Our results demonstrated that LVI has an unfavorable effect on OS and DFS in patients with early stage CRC. Moreover, the prognostic value was not altered by subgroup analysis based on district (Asian *vs*. non-Asian patients), LVI positive rate (≤15 *vs*. > 15 %), sample sizes (≤190 *vs*. > 200), tumor site (colon *vs*. rectum *vs*. colorectum) and study quality (≤6 *vs*. > 6). Postoperative adjuvant chemotherapy has been shown to prolong DFS or/and OS in some stage II patients [34, 37], besides NCCN guidelines recommend patients with high-risk stage II colorectal cancer should be considered for adjuvant chemotherapy. Thus it is reasonable to consider adjuvant chemotherapy for stage II patients in LVI positive status. The current NCCN Guidelines have not identified stage I patients who have a high risk of recurrence and this stage patients are not recommended for adjuvant chemotherapy. We suggest imposing stricter surveillance on stage I patients in LVI positive status.

Although Artac M et al. suggested that LVI was not an independent risk factor for survival [7]. A potential value of LVI may be useful in identifying tumors with occult lymph node metastasis [38, 39], for high-risk patients with node negative (stage I/II) tumors warranting adjuvant chemotherapy [32, 40]. As we known, 12 lymph nodes or more must be surgically resected to achieve accurate staging [41]. Lymph node harvest is influenced by many factors, such as the extent of surgical resection, recovery from the resected specimen, and counts of microscopic slides [42, 43]. Twelve-node harvest is sometimes difficult to achieve in daily surgical practice, thus will result in stage migration. Because LVI correlates well with the status of lymph node metastasis and disease staging [21, 44], which make it possible to be a supplement for those understaged patients.

The incidence of LVI reported in the present study ranged from 5.2% to 30.3%. The figure was significantly higher in non-Asian region than in Asian region (*p* < 0.05), and the difference in geographic setting had not been reported previously. The wide variation in LVI positive rates may due to many factors, including different geographic setting, differences in the characteristics of tumors, different criteria for LVI presence, and variations in the use of special stains or immunohistochemical (IHC) staining. Furthermore, the College of American Pathologists’ consensus statement did not recommend the use of any special stains or immunohistochemical stains to diagnose vessel invasion [45]. The status of LVI was mainly assessed by conventional hematoxylin and eosin (H&E) staining method in the included studies. One major challenge of this method is that the identification of LVI is subjective and inconsistency [46]. Interobserver variability in diagnosis of LVI was substantial on H&E slides and did not improve upon use of IHC staining for CD31 and D2-40 [47]. On the contrary, some investigators added IHC staining to improve accuracy rate [10, 48]. Another problem with H&E staining method is hard to distinguish lymphatic vessels invasion from blood vessel invasion (BVI) [46]. In the present study, we combined blood vessel invasion and lymphatic vessels invasion into lymphovascular invasion. We were aware that a study by Liang P reported that only lymphatic vessels invasion is associated with lymph node metastasis, and BVI is associated with distant recurrence in the manner of immunohistology [48].

Several potential limitations affect the results of this meta-analysis. First, all included studies were observational studies, and some of the sample sizes were relatively small. Second, several studies were excluded due to insufficient data to determine the correlation coefficients. One study included was unable to access full-text with sufficient data to calculate from abstract, but we could not get more detail information [9]. Finally, rare study compared the effect of adjuvant chemotherapy in the stage II patients with LVI positive status. More randomized controlled trials (RCTs) should be performed to validate the benefit form adjuvant chemotherapy for stage I/II CRC patients with LVI positive.

## MATERIALS AND METHODS

### Literature search

A systematic search of the PubMed, Web of Science, and Embase databases was performed to identify all relevant articles published up to 10 Dec 2016 with the limits of English. The following Medical Subject Heading (MeSH) terms or keywords were used: “colorectal neoplasms [MeSH Terms] OR colonic neoplasms [MeSH Terms] OR rectal neoplasms [MeSH Terms] ” AND “lymphovascular invasion OR lymphovascular permeation”. Moreover, we also check for potentially relevant studies through screening the references of the relevant articles.

### Inclusion criteria

All studies were required to meet the following criteria: (1) the diagnosis of CRC and LVI were based on pathological examination (2) the assessment of the relationships between PVI and the prognosis of CRC patients with stage I/II was reported with overall survival (OS) or/and disease-free survival (DFS), and (3) a hazard ratio (HR) was reported with 95% confidence interval (CI) or had sufficient data to estimate the HR and 95 % CI if not directly presented. When results reported from the same patient population, the most recent study or the largest dataset was included.

Abstracts and reports from meetings were excluded. Articles in which the outcomes of interest were not reported or from which it was impossible to calculate outcomes from the original data were also excluded.

### Data extraction and quality assessment

Two authors (H. Yuan and J.B. Xu) independently reviewed each eligible study and extracted the data. If any disagreements existed, they were resolved by discussion. Data retrieved from the articles included the first author's name, publication year, patient characteristics(number, sex, age, duration of follow-up, community), tumor site, LVI positive rate, study design, TNM staging, treatment characteristics [neoadjuvant chemoradiotherapy (nCRT) and postoperative chemoradiotherapy (pCRT)], and outcomes (OS and DFS). The quality assessment of including studies was based on the criteria of the Newcastle-Ottawa Quality Assessment scale (NOS) [49]. The study with NOS scores > 6 was regarded as high-quality studies.

### Statistical analysis

Meta-analysis was performed in line with the PRISMA guidelines [50]. Pool meta-analysis for OS/DFS was performed by using the Review Manager 5.3 software. The pooled HR and 95 % CI were calculated using the method of inverse variance and the *P* value threshold was set at 0.05. Heterogeneity was assessed by a chi-square-based Q statistical test and the *I*2 value. When *P* was < 0.10 or/and the *I*2 value was > 50 %, it meant significant heterogeneity between the studies and a random-effect model could be used; otherwise, a fixed-effect model was used [51]. Subgroup analysis, sensitivity analysis, publication bias and meta regression were performed using STATA 12.0 software.

Publication bias was assessed using a funnel plot. Subgroup analyses were performed by geographic setting, treatment, TNM staging, tumor site, LVI positive rate, and study quality. The difference of the incidence of LVI between in non-Asian region and in Asian region was performed by independent T-test.

## CONCLUSIONS

In conclusion, this meta-analysis indicated that LVI is a poor prognostic factor for stage I/II CRC patients. Stage II patients with LVI positive should be considered for treatment with effective adjuvant therapies, and stricter surveillance may be imposed on stage I patients in LVI positive status.

## Abbreviations

LVI: (lymphovascular invasion)
CRC: (colorectal cancer)
OS: (overall survival)
DFS: (disease free survival)
HR: (hazard ratio)
CI: (95% confidence interval)
